# New and old complex recombinant HIV-1 strains among patients with primary infection in 1996–2006 in France: The French ANRS CO06 primo cohort study

**DOI:** 10.1186/1742-4690-5-69

**Published:** 2008-08-01

**Authors:** Pierre Frange, Julie Galimand, Nicole Vidal, Cécile Goujard, Christiane Deveau, Faouzi Souala, Martine Peeters, Laurence Meyer, Christine Rouzioux, Marie-Laure Chaix

**Affiliations:** 1 EA 3620, Université Paris – Descartes, Laboratoire de Virologie, Hôpital Necker – Enfants Malades, AP-HP, Paris, France; 2 UMR145, Institut de Recherches pour le Développement (IRD) et Université Montpellier 1, Montpellier, France; 3 Département de Médecine Interne et de Maladies Infectieuses, Hôpital Bicêtre, AP-HP, Le Kremlin – Bicêtre, France; 4 Institut National de la Santé et la Recherche Médicale (INSERM) U822, Le Kremlin-Bicêtre, France; 5 Université Paris Sud, Faculté de Médecine Paris-Sud, Le Kremlin-Bicêtre, France; 6 Service d'Epidémiologie et de Santé publique, Hôpital Bicêtre, AP-HP, Le Kremlin Bicêtre, France; 7 Département de Maladies Infectieuses, Hôpital Pontchaillou Hospital, Rennes, France

## Abstract

**Background:**

Prevalence of HIV-1 non-B subtypes has increased overtime in patients diagnosed at the time of primary infection (PHI) in France. Our objective was to characterize in detail non-B strains which could not be genetically classified into the known subtypes/Circulating Recombinant Forms (CRFs).

**Methods:**

Among 744 patients enrolled in the ANRS PRIMO Cohort since 1996, 176 (23.7%) were infected with HIV-1 non-B strains. The subtype/CRF could not be identified in RT for 15 (2%). The V3-V5 *env *region was sequenced and 3 strains (04FR-KZS, 06FR-CRN, 04FR-AUK) were full-length sequenced. Phylogenetic and bootscan analyses were used to characterize the mosaic structures.

**Results:**

Among V3-V5 sequences, 6 were divergent A, 2 distantly related to E or D, 2 C, 1 B and 2 remained unclassified. 04FR-KZS, isolated in a Congolese woman infected in France, clustered with 2 previously described viruses from the Democratic Republic of Congo. They represent CRF27_cpx involving A/E/G/H/J/K/U subtypes. 06FR-CRN, isolated in a homosexual Caucasian patient, was a B/C/U recombinant involving a Brazilian C strain. 04FR-AUK, isolated in a Congolese patient infected in France, was a A/K/CRF09/U recombinant clustering from *gag *to *vif *with HIV-1 MAL. Others PHI were further observed in 2006–2007 with 1 KZS and 5 CRN-like viruses, suggesting their spread in France.

**Conclusion:**

This study illustrates the increasing HIV-1 diversity in France associating new (06FR-CRN) and old (CRF27_cpx and "MAL-like" 04FR-AUK) strains, which are rare in their region of origin but may have a possible founder effect in France. Our results strengthen the French guidelines recommending viro-epidemiological surveillance of HIV-1 diversity.

## Background

Human Immunodeficiency Virus type 1 (HIV-1) viruses are characterized by extensive genetic diversity driven by the error-prone reverse transcriptase (RT) enzyme in the context of rapid viral turn-over and its highly recombigenic nature [1,2]. HIV-1 variants are classified in three major phylogenetic groups: M (main), O (outlier) and N (non-M/non-O) each corresponding to independent cross-species transmissions with SIVs from wild chimpanzees and/or gorillas in West Central Africa [3]. Only group M viruses have spread across Africa and to all other continents. Group M can be further subdivided into subtypes (A-D, F-H, J-K) and sub-subtypes (A1-A4, F1-F2). In addition an increasing number of Circulating Recombinant Forms (CRFs, CRF01-CRF37) and many Unique Recombinants Forms (URFs) have also been described [4-6]. The geographic distribution of the different HIV-1 M variants is very heterogeneous and specific distributions of the various subtypes are seen among the different continents, even from country to country or within countries [7]. In France, subtype B predominates, like in other European countries and in North America, but the overall prevalence of non-B strains is increasing, also among French Caucasian individuals [8,9]. In chronically newly diagnosed HIV-1 infections, non-B strains represented 10% of the cases in 1998, 33% in 2001 and 50% in 2005 [10]. The distribution of HIV-1 strains circulating in France is particular, as successive migratory flows from African countries with French language have led to an exceptional viral diversity, higher than in other countries where subtype B epidemic is predominant. The increasing diversity may have implications for HIV-1 diagnosis, treatment, drug resistance, vaccine development, transmission and pathogenesis.

The French multicenter PRIMO Cohort study ANRS CO06 started in 1996 and contributed to the epidemiological surveillance of viral strains acquired at the time of PHI: the frequency of non-B strains increased from 10% in 1998–1999 to 28% in 2006 [11]. This result is similar to the frequency described in recently infected patients included in the European SPREAD study (20% of non-B viruses) [12]. In the PRIMO Cohort, 15 non-B strains, which could not be classified into any of the known subtypes or CRFs after RT phylogenetic analysis, have also been observed since 1996. The objective of our study was to characterize more in detail these strains. Phylogenetic analysis of their V3-V5 *env *region has been performed and 3 of them were full-length sequenced, as they seemed particularly divergent.

## Methods

### Study population

The study population comprised 768 patients presenting with PHI enrolled in the French PRIMO Cohort study between November 1996 and October 2006 [13]. The enrolment criteria were: (i) a negative or indeterminate HIV enzyme-linked immunosorbent assay associated with a positive antigenemia or plama HIV RNA; (ii) a Western blot profile compatible with ongoing seroconversion (incomplete Western blot with an absence of antibodies to *pol *proteins); or (iii) an initially negative test for HIV antibody followed within 6 months by a positive HIV serology. For all patients, plasma and peripheral blood mononuclear cells (PBMCs) samples were collected at inclusion and stored. Subsequent viral genotypic drug resistance testing and HIV-1 subtyping were systematically performed.

### V3-V5 env sequences

DNA was extracted from PBMCs with the QIAamp^® ^DNA Mini Kit (Qiagen SA, Courtaboeuf, France). *Env *(640 bp) fragments were amplified by ED3/ED12 as outer and ES7/ES8 or Env7/ED33 as inner primers [14] with the Expand High Fidelity plus PCR System^® ^according to the instructions of the manufacturer (Roche Applied Science, Mannheim, Germany). We used PCR conditions as previously described [14]. The amplified products were purified with QIAquick PCR Purification Kit^® ^(Qiagen SA, Courtaboeuf, France). Nucleotide sequences were obtained by direct sequencing of the amplified DNA using the inner primers and Big Dye Terminator V1.1^® ^technology (Applied Biosystems, Foster City, CA, USA). Electophoresis and data collection were performed on an ABI 3130 Genetic Analyser^® ^sequencer (Applied Biosystems, Foster City, CA, USA).

### Full-length genome sequences

Three overlapping nested PCRs were done to obtain the sequence of entire genomes. A fragment that included accessory genes, the entire envelope and *nef *was amplified with hpol4235 and LsiGI as outer primers (~5 kb). *Gag *and *pol *genes were amplified with G00 and hpol4538 as outer primers (~4.2 kb). Unintegrated circular DNA was targeted to amplify the rest of *gag *and *LTR *with Env1 and G00rev as outer primers (~5 kb) [15]. Several primers were subsequently used to perform nested PCR into these amplified fragments, as previously described [15]. PCR and sequence primers are available upon request. The Expand Long Template PCR System^® ^Taq polymerase (Roche Applied Science, Mannheim, Germany) was used according to the instructions of the manufacturer and PCR conditions were used as previously described [15]. The amplified products were purified using a QIAquick Gel Purification Kit^®^(Qiagen SA, Courtaboeuf, France) and sequenced as described above. Sequences were obtained for both DNA strands and contigs were assembled and edited using the Sequence Navigator^® ^software [16].

### Phylogenetic tree analysis

Phylogenetic relationships of the RT, V3-V5 and full-length genome sequences were estimated from sequence comparisons with previously reported representatives of group M subtypes and sub-subtypes. In addition to pure subtypes, we analyzed the new strains also for their phylogenetic relationship to CRFs for which sequences are available in the HIV database or genbank . For the analysis of the 04FR-KZS and 04FR-AUK viruses, isolated in patients born in the Democratic Republic of Congo (DRC), we added reference strains of CRFs circulating in Africa (CRF01_AE, CRF02_AG, CRF04_cpx, CRF05_DF, CRF06_cpx, CRF09_cpx, CRF11_cpx, CRF13_cpx, CRF18_cpx and CRF19_cpx) and sequences from previously reported divergent strains (tentative subtype L [17], and complex URFs from Central Africa [18,19]). For the analysis of the 06FR-CRN virus, isolated in a Caucasian French patient and clustering with subtype C in the V3-V5 region, we included additional subtype B, C and D strains from different geographic areas as well as CRFs harbouring subtype C fragments: CRF07_BC, CRF08_BC, CRF10_CD and CRF31_BC. The *env *and full-length nucleotide sequences were aligned using Clustal W (v1.7) [20] with minor manual adjustments. Phylogenetic trees were constructed with the neighbor joining method, and reliability of the branching orders was implemented by Clustal W using the boostrap approach. TreeView Win16 [21] was used to draw trees for illustrations. Genetic distances were calculated using the Kimura two-parameter method, with a transition weight of 0.5.

### Analysis for intersubtype mosaicism

To analyze the recombinant structure of the new viruses, several additional analyses were performed. The Simplot 3.5.1 software was used to determine the percentage of similarity between selected pairs of sequences and to calculate bootscan plots, by performing bootscanning on parsimony trees using SEQBOOT, DNADIST (with Kimura's two-parameter method and F84 model of maximum likelihood method, transition/transversion ratio = 2.0), NEIGHBOR and CONSENSE from the Phylip package [22]. In the similarity and bootscan plots, the new sequences were compared with consensus sequences (50% threshold) of the non-recombinant subtypes and some CRF reference strains. The regions that did not cluster with any of the known subtypes were submitted to BLAST analysis (BLASTN 2.0.6 on line; ), to see whether they are closely related with previously described unknown fragments of other HIV-1 strains.

### Nucleotide sequences

Full-length sequences of 04FR-KZS, 04FR-AUK and 06FR-CRN strains were submitted to GenBank with the following accession numbers: – [GenBank:AM851091, -GenBank:EU448295 and -GenBank:EU448296], respectively.

## Results

### Characteristics of the study population

**Figure 1:**
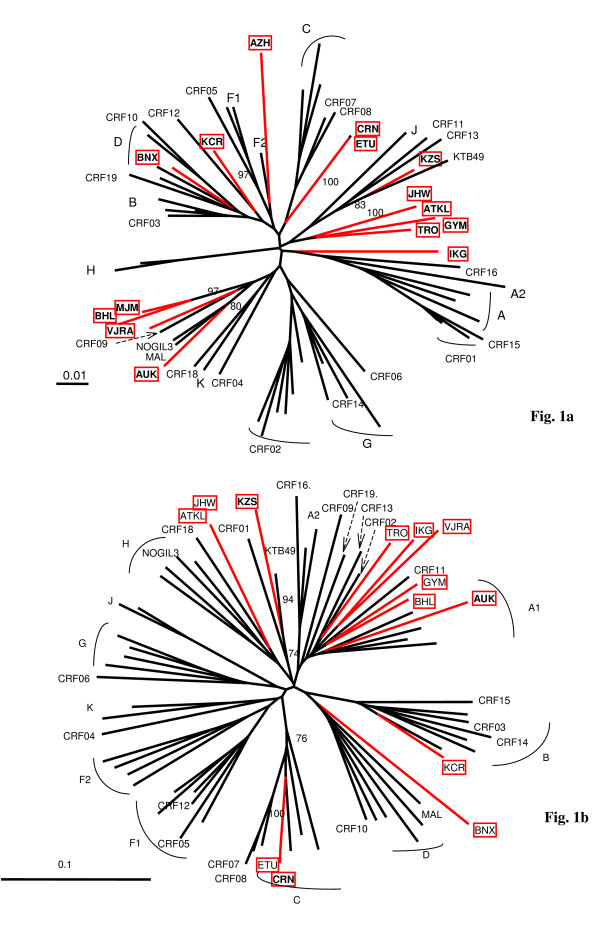
**Phylogenetic tree analysis of the 15 "undetermined" viruses isolated in patients enrolled at the time of primary infection**. In the phylogenetic trees, based on the RT nucleotide sequence of the 15 “undetermined” strains (a) and the corresponding V3-V5 env region for 13 of them (b), the reference sequences were as follows: 3 references for all pure subtypes, 1 reference for 18 previously described CRF (CRF01-16 and CRF18-19), and unique recombinant MAL [23], NOGIL [24] and 97CD-KTB49 [18] strains. Trees based on unambiguously aligned nucleotides were generated by the neighbour-joining method, and the reliability of each clustering was assessed by bootstrapping with one hundred replicates implemented by Clustal W. The Simplot v3.5.1 performed bootscanning on NJ trees along the genome alignment by moving a 400 base pairs window along the genome alignment with 20 base pairs increment and one hundred replicates for each phylogeny. Only bootstrap values above 70 at each of the internal branches defining a subtype are shown.

From 1996 to October 2006, 744 strains have been genetically characterized among the 768 patients recruited in the PRIMO cohort. Phylogenetic analysis revealed that 176 (23.7%) were HIV-1 non-B strains. Whereas the majority of them (57%) were CRF02_AG viruses [11], 15 (2%) were not classified into the known HIV-1 subtypes or CRFs (Figure 1a). The clinical, virological and immunological characteristics of the 15 patients at the time of their inclusion in the cohort are summarized in the Table 1. Among the 15 strains, two clusters of very closely related strains (> 99% homology) were identified: 03FR-ATKL and 03FR-JHW from 2 heterosexual partners, and 06FR-CRN and 06FR-ETU among two men having sex with men (MSM) whose PHI occurred in 2006, although diagnosed in two different cities. V3-V5 sequences were done on the 15 unclassified samples. A total of 13 "undetermined" strains have been amplified and sequenced: 6 clustered in the subtype A radiation but did not form a well supported cluster with an A sub-subtype or CRF specific subtype A lineage. Two samples were distantly related to subtype E or D, 2 could be classified as C, 1 as B and 2 remained undetermined (Figure 1b).

**Table 1:** Characteristics of the 15 patients with primary HIV-1 infection with an undetermined strain at the time of their inclusion in the PRIMO Cohort.

Patient	Sex	Year of birth	Country of birth	Primoinfection			Baseline characteristics		
				Country of infection	Year	Mode of infection	CDC stage	CD4 count (/mm^3^)	HIV RNA load * (log_10 _cp/ml)
MJM	F	1970	Algeria	France	1996	heterosexual	A	888	4.50
BNX	M	1969	France	France	1997	homosexual	A	507	4.60
TRO	F	1939	France	France	1999	heterosexual	A	932	5.50
BHL~	M	1947	France	Togo	2001	heterosexual	A	1078	5.60
GYM	F	1972	CAR	France	2002	heterosexual	A	211	5.71
AZH	F	1960	France	France	2002	heterosexual	A	553	3.97
IKG	M	1948	France	France	2002	heterosexual	A	436	5.39
VJRA	M	1976	Cameroon	France	2002	heterosexual	A	299	5.08
JHW	M	1966	CAR	France	2003	unknown	A	359	5.13
ATKL	F	1964	France	France	2003	heterosexual	A	359	4.02
AUK§	F	1975	DRC	France	2004	heterosexual	A	555	3.54
KZS§	F	1981	DRC	France	2004	heterosexual	A	196	5.21
CRN§	M	1967	France	France	2006	homosexual	A	240	6.30
ETU	M	1973	France	France	2006	homosexual	A	128	5.56
KCR	M	1967	France	France	2006	homosexual	A	440	5.44

(DRC = Democratic Republic of Congo, CAR = Central African Republic).

~Caucasian patient likely to have been infected in Sub-Saharian Africa.

* HIV-1 RNA viral load performed using Cobas Amplicor HIV-1 Monitor v1.5 test^® ^(Roche diagnostic System, Alameda, CA).

§Patient whose recombinant virus has been full-length sequenced.

### Full-length genome sequencing of 3 HIV-1 strains

**Figure 2:**
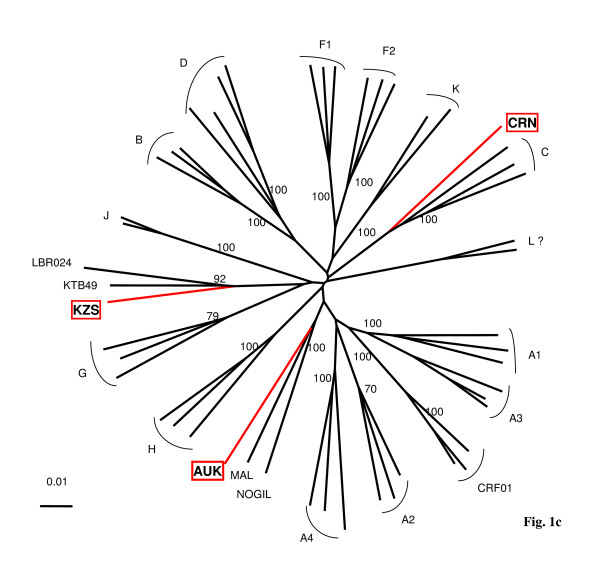
**Phylogenetic tree analysis of the 3 full-length sequenced strains (04FR-KZS, 06FR-CRN, 04FR-AUK).**The sequences were aligned with HIV-1 subtype and subsubtype references, as well as CRF01_AE, MAL, NOGIL, 97CD-KTB49 and 02CD-LBR024 [19] sequences.

To study more in detail these divergent HIV-1 strains, we characterized the full-length genome for 04FR-KZS, 06FR-CRN and 04FR-AUK strains. They were chosen for different reasons: 04FR-KZS strongly clustered with a previously described complex recombinant virus (97CD-KTB49 [18]); 06FR-CRN was undetermined in RT phylogenetic analysis but strongly clustered in the subtype C in V3-V5 analysis; 04FR-AUK displayed extensive similarity in the RT region with one of the earliest African HIV-1 strains, MAL, previously described as an A/D/K/U recombinant virus [23]. Moreover these 3 strains circulated in France between 2004 and 2006. The patients infected with these strains were diagnosed soon after infection with an acute retroviral syndrome (estimated delay from infection: 20, 24 and 22 days, respectively). Two of them (04FR-KZS and 06FR-CRN) presented with a very low CD4 cell count and a high viral load; the third one (04FR-AUK) had a moderate CD4 cell count decrease associated with a spontaneously low viral load (Table 1). The 3 new full-length sequences were compared with representatives of all subtypes, sub-subtypes, CRFs available in the HIV database and with other unpublished and published URFs. The phylogenetic tree analysis (Figure 2) showed that 06FR-CRN formed a well supported cluster with subtype C. The 04FR-AUK strain did not cluster with any known sequence but seemed to be related to the previous reported complex recombinant strains from central African origin: MAL [23] and NOGIL (A/K/H/U) [24] which have a common A/K/U structure in *gag*-*pol*.

### Analysis of 04FR-KZS recombinant structure

**Figure 3:**
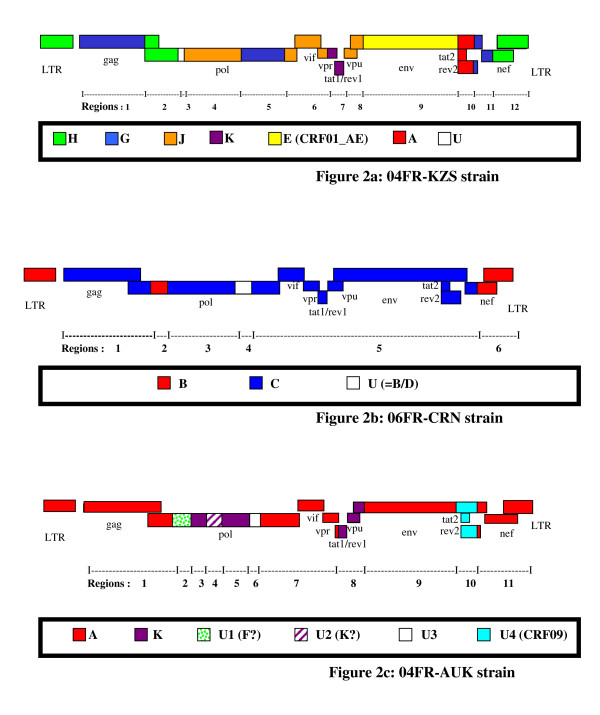
**Schematic representation of the subtype pattern of the 04FR-KZS (a), 06FR-CRN (b) and 04FR-AUK (c) strains**. U = unclassified region.

04FR-KZS formed a separate subcluster with 2 previously characterized *env *subtype E isolates from DRC with a recombinant structure different from CRF01-AE (97CD-KTB49 and 02CD-LBR024) and has been recently described as CRF27cpx, involving six different HIV-1 subtypes (A, E, G, H, J, K) and a small unclassified fragment (Fig. 3a) [19]. The 04FR-AUK and 06FR-CRN strains were subjected to further analysis in order to determine their exact structure.

### Analysis of 06FR-CRN recombinant structure

**Figure 4:**
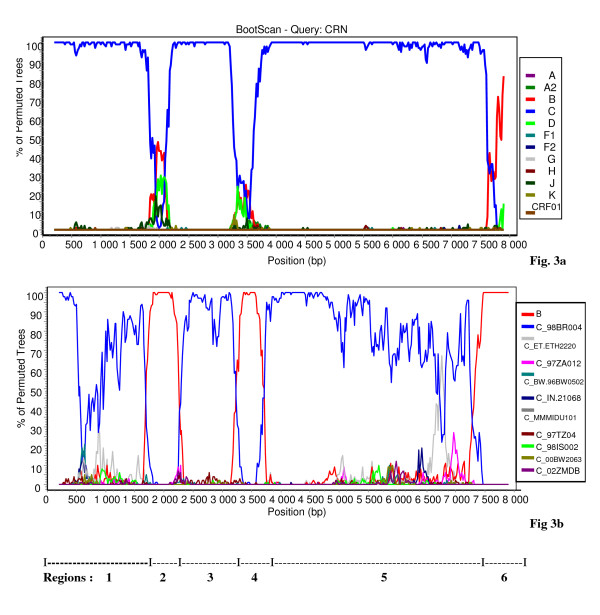
**Analysis of the recombinant structure of 06FR-CRN strain**. Bootscan plots (a) showing the complex mosaic structure of the 06FR-CRN strain (9684bp). The full-length sequence was aligned with HIV-1 subtype and subsubtype reference sequences (gaps were stripped from the 8116 unambigously aligned base pairs). (b) Bootscan plots with B and C references performed to better characterize the geographic origin of the 06FR-CRN strain.

**Figure 5:**
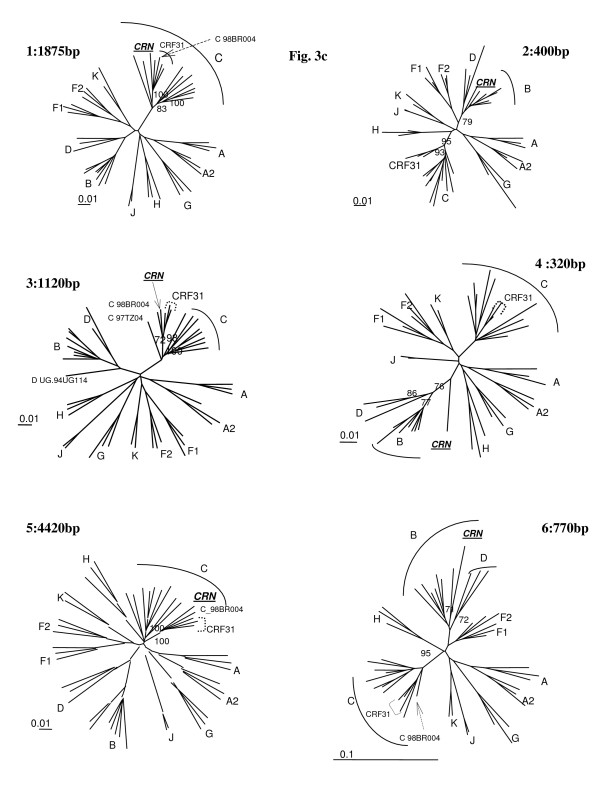
Phylogenetic tree analysis of each of the 6 recombinant regions of 06FR-CRN strain defined in Fig. 6b and represented in schema 2b.

The bootscan analysis of 06FR-CRN (Fig. 4a) showed that the majority of the genome is subtype C except two small regions in *pol*: the first one included part of the RT gene and clustered with subtype B; the second one (5'end of the *integrase *gene) clustered with the common branch for B and D subtypes (region 4). The 3'end of the *nef *gene and *LTR *were subtype B. Figure 4b shows a more detailed bootscan analysis of 06FR-CRN against 10 subtype C reference strains, isolated from different regions over the world and illustrates that 06FR-CRN "C" regions strongly clustered with 98BR-BR004, isolated in Brazil [25]. Despite iterative bootscan analysis including additional B and D reference strains or with BLAST analysis, the region 4 remained undetermined. The subtype identifications of the various genomic regions were all confirmed by phylogenetic tree analysis of the corresponding fragments (Figure 5). We included reference sequences of CRF31_BC strains in these trees to illustrate the differences between this previously described CRF and 06FR-CRN, which both resulted from the recombination between a Brazilian C strain and a B virus. Figure 3b shows the overall mosaic structure of the new B/C/U 06FR-CRN strain.

### Analysis of 04FR-AUK recombinant structure

**Figure 6:**
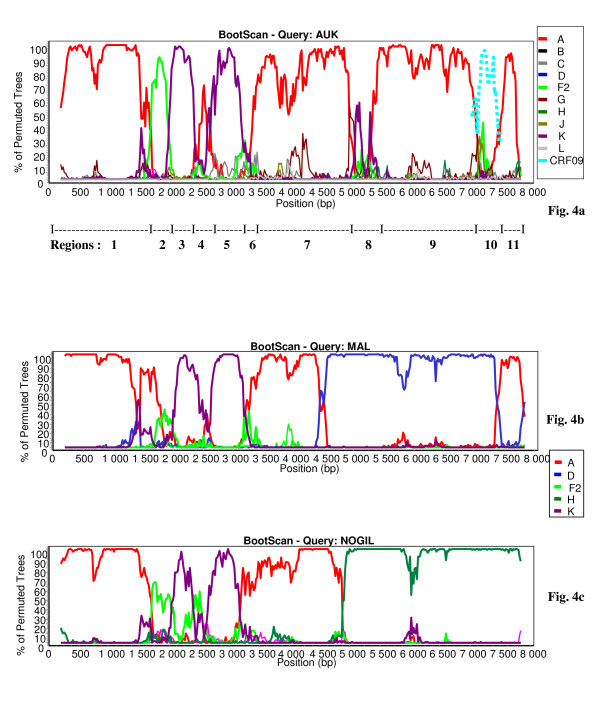
**Analysis of the recombinant structure of 04FR-AUK strain**. Bootscan plots (a) showing the complex mosaic structure of the AUK strain (9680bp). The full-length sequence was aligned with HIV-1 subtype and subsubtype reference sequences (gaps were stripped from the 8051 unambigously aligned base pairs). The same analysis was then performed in the undetermined region 10 by adding CRF09_cpx reference sequences (in doted lines). Bootscan plots showing the mosaic structure of the previously reported MAL [23] (b) and NOGIL [24] (c) strains.

Although subtype A and K predominate, the complexity of the new 04FR-AUK strain was readily apparent from the similarity plots (data not shown) and the bootscan analyses (Fig 6a). The *LTR*, gag, *vif, vpr *and *nef *genes and the majority of *pol *and *env *were subtype A (regions 1, 7, 9, 11). A short region, located at the 5'end of the RT, clustered with the common branch for F1 and F2 sub-subtypes in the phylogenetic analysis although not with a significant boostrap value (region 2). Therefore we classified this region as "undetermined" (U1), but it might represent an "F" variant. The *pol *gene included two regions (regions 3 and 5) which were clearly subtype K. A small region between them (region 4) was not well defined in the bootscan analysis and was therefore named "undetermined" (U2). However, 04FR-AUK clustered with subtype K in the phylogenetic tree analysis of this region, and may be considered as a divergent "K". In the 5' end of the *integrase*, a small region could not be clearly defined in the bootscan and phylogenetic tree analyses and was therefore classified as undetermined (region 6 = U3). The 3' end of the accessory gene region, including the entire *vpu *gene, was subtype K (region 8). On the boostrap and similarity plots, a 350 bp region at the 3'end of the *env *gene seemed difficult to classify (region 10 = U4). To better characterize the undetermined regions, we performed a BLAST search. The best match (94%) was found with an "undetermined" fragment of CRF09_cpx in region 10 only. We included in the bootscan analysis (figure 6a) of this region CRF09 strains and subsequent phylogenetic tree analysis, confirmed that this 04FR-AUK clustered significantly with the U fragment from CRF09. Figure 3c shows the overall mosaïc structure of the new 04FR-AUK strain. Finally, this complex A/K/CRF09/U virus strongly clustered with MAL and NOGIL viruses from *gag *to *vif *in the phylogenetic tree analyses. The MAL/NOGIL and MAL/04FR-AUK divergence breakpoints were located at the same place in the *vif *gene, while the NOGIL/04FR-AUK divergence breakpoints were located in the *vpr *gene, as shown in the bootscan analyses of MAL and NOGIL (Fig. 6b and 6c). In the 3'end of the *nef *gene and the *LTR*, NOGIL was subtype H but 04FR-AUK and MAL clustered together again in subtype A. Overall, it can be stated that the 5'end of the MAL/NOGIL/04FR-AUK strains have a common parental ancestor.

**Figure 7:**
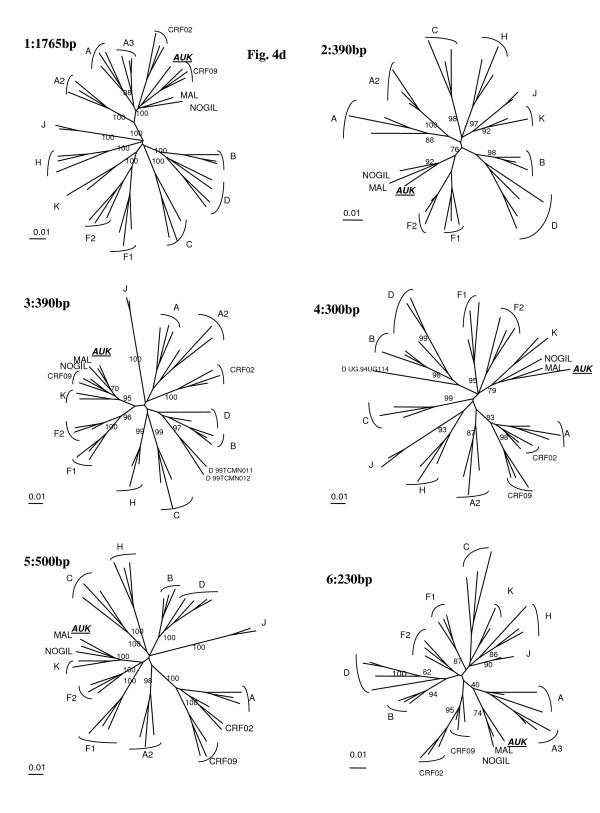
Part 1

**Figure 8:**
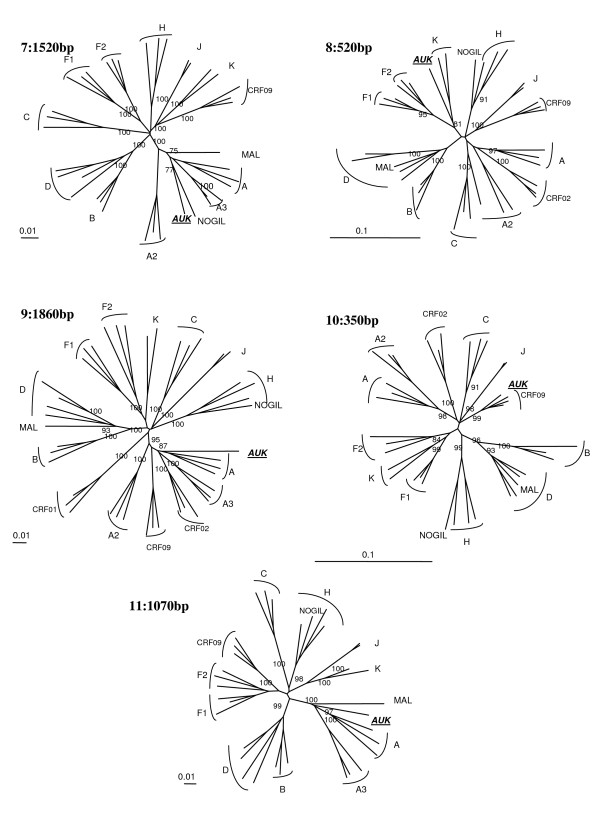
Part 2

To determine to what extend previously reported strains were related to MAL, NOGIL or 04FR-AUK strains, blast searches were done by cutting the full-length sequence into several fragments (about 1200–1300 bp), because an initial blast analysis with the complete genome sequence did not provide any significant information. We identified four strains from DRC (97CD-MBFE185, 97CD-MBS30, 02CD-KP061 and 02CD-KP097), one from Cameroon (97CM-MP814) and one from Senegal (98SN-40HALD) [26,27] for which partial *pol *sequences (1500 bp, *protease *and RT) clustered with 91% bootstrap values with 04FR-AUK/MAL/NOGIL and which displayed the same recombinant structure in that part of the genome as shown by additional bootscan and simplot analysis. Some of these strains had also been sequenced in other genomic regions such as the V3-V5 *env *or p24 *gag *region. Similarly as for 04FR-AUK, the following strains 97CD-MBFE185, 97CD-MBS30, 02CD-KP061 and 02CD-KP097 were also subtype A in the V3-V5 region, and 97CD-MBFE185, 97CD-MBS30 and 97CM-MP814 were also subtype A in *gag *p24. Moreover, other sequences from Gabon (97GA-G15, 97GA-ME56, 97GA-PP98, 97GA-TB64, 00GA-GAB22S, 97GA-G32, 97GA-TB45) [28], Congo (CgARV64 and CgARV64) [29] and Senegal (97SN-1055) [30] formed a subcluster with MAL/NOGIL/04FR-AUK supported by 78% bootstrap within subtype A. (See figures 7 and 8

## Discussion

In the French PRIMO Cohort study, which has been enrolling 744 patients presenting during PHI, 24% of the subjects were infected by a non-B strain over the 1996–2006 period; 2.02% (15/744) viruses could not be classified after phylogenetic analysis of the RT gene. The frequency of these unclassified strains remained stable from 1.30% in 1996–2001 to 2.51% in 2002–2006 (p = 0.3). These viruses were all isolated in patients whose PHI occurred in France, except for one patient (01TG-BHL) who acquired HIV in Togo: 10 HIV infections occurred in Caucasians. Sequence and phylogenetic analysis of V3-V5 region did not allow to classify the majority of these viruses.

In this report, we describe the full-length genome for 3 new HIV-1 non-B strains identified during PHI in France; these strains circulate among migrants but also in the Caucasian population. The 04FR-KZS strain was isolated in a Congolese patient whose PHI occurred in France in 2004 and has led to the recent characterization of CRF27_cpx, involving 6 subtypes (A/E/G/H/J/K) and 1 unclassified fragment [19]. The other representatives of this CRF, 97CD-KTB49 and 02CD-LBR024, derived from patients in DRC, were isolated 7 and 2 years earlier respectively among national sentinel serosurveillance studies.

06FR-CRN strain is an illustration of the increasing complexity of the global HIV-1 genetic diversity. 06FR-CRN represents actually a new URF between subtype B and a Brazilian subtype C. In addition, a short region, at the 5'end of the *integrase *gene, remained "undetermined" as it clustered with the common branch for B and D subtypes and could therefore not be clearly identified as B or D. Subtype B strains predominate in Brazil but, like in France, subtype C viruses have been recently introduced. Subsequently, recombinations between B and C led to CRF31_BC and multiple unique B/C recombinants. 06FR-CRN, a unique B/C/U recombinant involving a Brazilian subtype C, could have been exported from Brazil into France, or it could derive from a pure Brazilian subtype C that recombined in France or from a Brazilian B/C strain recombining with the U fragment in France.

04FR-AUK is a complex recombinant strain related to HIV-1 strains which have been circulating for a long time in Central Africa. The 5'end of 04FR-AUK is related to HIV-1 MAL which has been described in France more than 20 years ago in a Congolese patient [23], and to HIV-1 NOGIL, described in Norway [24]. The 04FR-AUK sequence contributed to better define the complex structure and the evolution of MAL and "MAL-like" NOGIL strains. Jonassen and col. have previously reported that MAL and NOGIL may be derived from a postulated MAL-NOGIL parental lineage, which probably existed before 1981 [24]. Our results showed that an additional strain is derived from this parental lineage. We showed that the 04FR-AUK/NOGIL divergence breakpoint was located in the *vpr *gene, while the divergence breakpoint between MAL and these "MAL-like" strains was located in the *vif *gene, earlier in the viral genome. Secondly, 04FR-AUK and MAL strongly clustered together in the *nef *gene and the LTR, whereas NOGIL belonged to a different subtype. Thus, it could be expected that 04FR-AUK derived from the recombination between the postulated parental lineage and another previously undescribed complex strain with a "A/K/U (CRF09)" *env *structure or that multiple independent recombination events occurred leading to the final observed structure. Subsequent screening of HIV-1 sequences available from genbank or Los Alamos database, for similarity with MAL, NOGIL and/or 04FR-AUK strains identified at least 6 strains which formed a well supported cluster with them in a 1500 bp fragment in *pol *(*protease *and RT). Moreover, further simplot and bootscan analyses confirmed the same recombinant structure in this part of the genome. In addition, for some of these strains, additional partial *env *and/or *gag *sequences were also available and were identified as subtype A similar as the 04FR-AUK strain, but different form MAL and NOGIL. However, full-length genome sequence will be necessary to identify whether these viruses share the same structure as 04FR-AUK. Interestingly, almost all these potential related viruses have their origin in central Africa, especially DRC or Cameroon, except one from Senegal.

Five other PHI have been further diagnosed since October 2006 with KZS and CRN-like viruses, suggesting their spread in France. Firstly, another CRF27 infection (06FR-BOR) was identified in a patient of the PRIMO Cohort based on partial *pol *(RT) and *env *(V3-V5) sequences. This PHI occurred in December 2006 in a 34-year old man, originating from DRC and infected in France after heterosexual intercourse. The genetic distance between 06FR-BOR and 04FR-KZS as well as epidemiological information, revealed absence of any epidemiological link. Interestingly, the genetic distances between the 4 reported CRF27 strains are relatively high (18% diversity in the *env *gene between 06FR-BOR and 04FR-KZS), indicating that CRF27-cpx is an old HIV-1 variant and that either several independent introductions occurred into France or either these viruses circulated for a longer period in France but remained undetected. Effectively, our network for the survey of the viral diversity in French primo-infected patients included each year only 5–10% of the overall estimated PHI [31]. This recent spread in France contrasts with the low (0.75%) and stable CRF27_cpx prevalence in Central Africa [32,33]. Secondly, four other strains have been isolated in our laboratory since November 2006 in French Caucasian MSM at the time of PHI, which -strongly clustered with 06FR-CRN and 06FR-ETU, with more than 99% homology among RT and V3-V5 sequences and seemed to belong also to the same contamination cluster (data not shown). These results suggest the recent and possible rapid spread of this URF in France which could become a CRF spreading in France among the population of MSM.

## Conclusion

Our study confirms the increasing complexity of HIV-1 viruses, not only in Central Africa [29], but also in France, even in Caucasian patients. This evolution is due to immigration flows from Africa, but also from South America and possible other regions, and associates new (06FR-CRN) and old (CRF27_cpx and "MAL-like" 04FR-AUK) HIV-1 strains, which are rare in their region of origin but may have a possible founder effect in France. Our results strengthen the French guidelines which recommend to perform genotypic resistance tests at the time of PHI to survey the frequency of resistant strains as well as the molecular epidemiological HIV-1 diversity.

## Competing interests

The authors declare that they have no competing interests.

## Authors' contributions

PF, JG, NV and MLC carried out the full-length sequencing and the phylogenetic analysis of the strains. CG, LM and CD carried out the coordination of the ANRS PRIMO cohort and have been involved in revising the manuscript critically. FS was the physician of the ANRS PRIMO cohort which carried out the medical follow-up of the included patient infected with the 06FR-CRN strain. MP and CR have been involved in revising the manuscript critically for important intellectual content and have given final approval of the version to be published.
